# Compact waste image classification with multi-student CNNs and edge-oriented model selection

**DOI:** 10.3389/frai.2026.1804734

**Published:** 2026-06-30

**Authors:** Mohamed Echchidmi, Anas Bouayad

**Affiliations:** Laboratory of Artificial Intelligence, Data Science, and Emerging Systems, Sidi Mohamed Ben Abdellah University, Fez, Morocco

**Keywords:** edge computing, knowledge distillation, model compression, sustainability, TinyML, waste classification

## Abstract

Automatic waste classification is an important enabling technology for cleaner cities, source-level recycling, and low-cost smart-bin systems. Although modern convolutional neural networks achieve strong recognition performance, their deployment on affordable edge devices remains constrained by memory footprint, computational cost, and response latency. This paper presents an edge-oriented compact CNN framework for waste image classification, combining a high-accuracy MobileNetV4 reference model with three lightweight student architectures: EfficientNet-Lite0, LCNet-0.5, and MobileNetV3-Small-0.5. All models are evaluated on TrashNet under a unified preprocessing, training, and size-accounting protocol, allowing a clear comparison of accuracy–efficiency trade-offs. On the main stratified train/validation/test split, the MobileNetV4 teacher achieves 97.09% top-1 accuracy, while the compact students retain strong performance with substantially smaller footprints: EfficientNet-Lite0 reaches 93.99% with 3.38 M parameters, LCNet-0.5 reaches 94.18% with only 0.61 M parameters, and MobileNetV3-Small-0.5 reaches 87.73% with 0.57 M parameters. A complementary stratified five-fold evaluation, including both knowledge-distilled and non-distilled student variants, provides a robust assessment of model behavior across data partitions and confirms LCNet-0.5 as the most suitable sub-megabyte candidate under the proposed size–accuracy selection rule. The selected LCNet-0.5 model achieves a macro-F1 score of 0.9247 on the main TrashNet test split and is integrated into a self-contained Raspberry Pi 3 Model B+ prototype that performs local camera-to-display inference with an observed end-to-end latency of approximately 1.0 s per image. Cross-dataset evaluation on RealWaste further shows that the compact model can be adapted effectively to cluttered real-world imagery through short fine-tuning. Overall, the results demonstrate that careful lightweight architecture selection, supported by knowledge distillation analysis and edge-prototype validation, can deliver accurate, compact, and practically deployable waste classifiers for resource-constrained environments.

## 1 Introduction

Municipal solid waste (MSW) is growing in both volume and complexity, and its management has become an environmental and public-health priority that cuts across the Sustainable Development Goals. Recent international assessments put global MSW generation at roughly 2.0–2.1 billion tons per year, with a projected rise to roughly 3.4–3.8 billion tons by 2050 under business-as-usual trends Kaza et al. (2018); UNEP (2024). The way that waste is handled is, if anything, an even larger problem than the volume: the World Bank reports that at least one-third of MSW is not managed safely, and open dumping remains the dominant disposal pathway in many lower-income settings Kaza et al. (2018). Material recovery rates also remain modest, with only a minority of MSW recycled or composted globally Kaza et al. (2018); UNEP (2024).

The recycling value chain has a clear bottleneck: sorting. Recovered materials must be separated with sufficient purity for downstream processing to be economically viable. In many low- and middle-income contexts, sorting is sustained by the informal sector, whose workers can achieve non-trivial recycling rates even when municipal infrastructure is limited Wilson et al. (2015); Medina (2008). Country-level reports paint the same picture. In Morocco, for example, the informal sector represents a substantial workforce involved in collection, sorting, and recycling, and scaling up formal sorting centers remains a known challenge World Bank IEG (2021). Practical sorting aids that work in resource-constrained settings would clearly help.

Computer vision has been a natural fit for this problem. Deep convolutional networks now classify recyclable materials from images with high accuracy Thielmann et al. (2025); Islam et al. (2025), and recent cross-validated benchmarks of CNN families on urban-waste imagery report top-1 accuracies above 99% (Sürücü and Ecemiş 2023). The catch is deployment cost. Even mobile-oriented families such as MobileNet and EfficientNet typically run to several million parameters and to compute and memory budgets that are awkward on low-cost embedded platforms Singh and Gill (2023); Cordova-Cardenas et al. (2025). This is particularly true for TinyML-class targets, where the available flash budget can be well-below a few megabytes Heydari and Mahmoud (2025).

There is a recurring tension between accuracy and size. Larger backbones reach strong accuracy on TrashNet, but they are overparameterized for memory-constrained use; shrinking depth or width directly can cause accuracy drops, especially on small datasets. Knowledge distillation (KD) is one possible training strategy for compact models, because a high-capacity teacher can provide softened class distributions in addition to hard labels. However, KD should not be assumed to improve performance automatically; its benefit depends on architecture, data regime, training budget, temperature, and the mixing coefficient between supervised and distillation losses. For this reason, KD is treated here as an evaluated component rather than as a guaranteed source of improvement.

Three methodological limitations are common in prior waste-classification work that uses compact architectures. First, efficiency is rarely reported using a unified set of objective indicators: parameter count, FP32 and estimated INT8 footprint, on-disk checkpoint size, and device-level latency. Second, compact models are usually evaluated in isolation rather than as part of a controlled, multi-candidate comparison under the same training protocol. Third, single train–test splits are still common, even though small and imbalanced datasets such as TrashNet can produce fold-sensitive estimates of macro-F1. The gap addressed in this paper is, therefore, methodological: a multi-student comparison that combines compact architecture selection, KD/no-KD cross-validation, explicit size accounting, domain-shift analysis, and Raspberry Pi prototype integration.

Concretely, we train a MobileNetV4 model (Qin et al., 2025) on TrashNet as a high-accuracy reference and evaluate three compact students—EfficientNet-Lite0, LCNet-0.5 (Cui et al., 2021), and MobileNetV3-Small-0.5—all designed for mobile or embedded use. The main single-split study reports KD-trained students under a 100-epoch budget. A complementary five-fold cross-validation study, run with a shorter training budget, compares KD and no-KD variants of the same student architectures. This distinction is important: the single-split and cross-validation numbers are not treated as identical training regimes, and the cross-validation evidence is used primarily to test ranking stability and the KD/no-KD trend. The selected LCNet-0.5 student is integrated into a physical prototype built around a Raspberry Pi 3 Model B+, where the observed end-to-end latency of the camera-to-display workflow is approximately 1.0 s per image.

The contributions of this work are:

- A controlled comparison of one high-accuracy MobileNetV4 reference model and three compact CNN students for TrashNet waste classification under shared preprocessing, optimization, and size-accounting settings.
- A conservative KD/no-KD cross-validation analysis showing that KD is competitive but not statistically superior in macro-F1 under the tested five-fold budget; this prevents overstating KD as the demonstrated source of improvement.
- A lightweight size–accuracy selection rule that identifies LCNet-0.5 as the best sub-megabyte candidate, with macro-F1 of 0.9247 on the main TrashNet test split.
- A Raspberry Pi 3 Model B+ prototype that embeds the selected LCNet-0.5 student in a practical camera-based waste-sorting workflow, with observed end-to-end latency of approximately 1.0 s per image and with explicit acknowledgment that full power/energy benchmarking remains future work.

## 2 Related work

### 2.1 CNNs for waste classification

Public datasets such as TrashNet Thung and Yang (2016) have made supervised learning for material recognition from images routine. Early studies used shallow CNNs or straightforward transfer learning from ImageNet-pretrained backbones Poudel and Poudyal (2022); Zhang et al. (2021), and the field then moved to deeper backbones such as VGG, ResNet, and MobileNet, with corresponding gains in accuracy but with millions of parameters and significant compute Ahmed et al. (2023); Fu et al. (2021). More recently, cross-validated benchmarks of several CNN families on urban-waste imagery have reported top-1 accuracies above 99% for MobileNetV2-class models trained with transfer learning and five-fold cross-validation (Sürücü and Ecemiş 2023), illustrating how mature the in-distribution accuracy of these models has become.

A parallel line of work targets compactness directly. WasteNet Vo et al. (2019) and compressed MobileNet variants Handhayani and Hendryli (2023) show that lightweight backbones can approach real-time performance with relatively modest resource use. Maintaining accuracy under strict memory and latency constraints is still difficult, particularly on CPU-only edge devices and low-cost smartphones. Our work continues this line by taking a strong MobileNetV4 backbone as a reference model and systematically evaluating three mobile-oriented students, with a deliberate focus on the accuracy–size trade-offs that matter on edge hardware. KD is evaluated as one training strategy, but the paper does not claim that it is statistically superior unless the results support that conclusion.

To clarify the distinction from prior efficiency-oriented studies, Table 1 summarizes representative works in terms of dataset, backbone, efficiency strategy, and reported accuracy and complexity. Most existing studies propose or fine-tune a single compact architecture for a given dataset and report performance with non-uniform efficiency indicators. The present work, in contrast, adopts a multi-student strategy: a single MobileNetV4 reference model and several mobile-friendly students are evaluated under controlled protocols, including KD and no-KD variants in cross-validation. This enables a more reproducible reading of the accuracy–efficiency curve on TrashNet while avoiding unsupported claims about KD superiority.

**Table 1:** Representative lightweight or efficiency-oriented waste-classification studies (NR, not reported).

Study	Dataset	Backbone	Efficiency strategy	Key result(s)	Difference vs. this work
Bircanoglu et al. (2018)	Study-specific	DenseNet121; RecycleNet	Architecture optimization	~7M → ~3M params; up to 95% acc.	No teacher/KD; single-model focus
White et al. (2020)	TrashNet (6)	WasteNet	Edge CNN design	97% acc.; Jetson Nano	No teacher/KD; no multi-student comparison
Zheng et al. (2023)	WasteImage	Focus-RCNet	Lightweight net; FLOPs	92% acc.; 418.8M FLOPs	No teacher/KD; no student-selection rule
Yong et al. (2023)	Domestic (4)	MobileNetV2	Lightweight TL	82.92% acc.	No KD; single backbone
Majchrowska et al. (2022)	Classify-waste (7)	EffNet-B2 (cls)	Pipeline/training	~75% cls. acc.	Different benchmark; not KD multi-student
(Sürücü and Ecemiş 2023)	TrashNet-style (6)	MobileNetV2 + 5 others	Five-fold CV; transfer learning	up to 99.36% acc. (MobileNetV2)	Larger backbone; no KD; no edge target

Numbers in parentheses in the Dataset column denote the number of waste classes in each dataset; *NR* = not reported.

### 2.2 Knowledge distillation

Knowledge distillation was introduced by Hinton et al. (2015) as a teacher–student framework in which a smaller model learns from a teacher's softened output distribution in addition to the hard labels. Subsequent work extended the idea in several directions, including intermediate feature regression Romero et al. (2015), attention transfer Zagoruyko and Komodakis (2017), and broader surveys and refinements aimed at model compression and edge deployment Gou et al. (2021). Multi-teacher and mutual-learning variants have also been studied Shang et al. (2023); Zhang Y. et al. (2018); our work uses a standard single-teacher setup, which keeps the protocol simple and the comparison across students clean.

For embedded vision, KD is a natural complement to lightweight architectures and quantization, but its empirical benefit must be checked against no-KD baselines. In our setup, the teacher is a MobileNetV4 model, the students are three mobile-oriented backbones, and KD-trained variants use a combination of label-smoothed cross-entropy and a Kullback–Leibler distillation term. The resulting models are compared along a size–accuracy spectrum, and the cross-validation analysis explicitly tests whether KD improves macro-F1 relative to the same student architectures trained without KD.

### 2.3 Model compression

Model compression techniques typically aim to reduce storage and compute while preserving accuracy. Pruning removes redundant weights or channels Han et al. (2016); He et al. (2017); quantization maps floating-point parameters to low-bit integer formats such as INT8 Jacob et al. (2018); Krishnamoorthi (2018). Several studies combine KD with compression to compensate for the accuracy loss caused by aggressive reductions Polino et al. (2018). Orthogonal to these methods, architectures built on depthwise-separable convolutions provide a strong structural prior for compactness. Our approach combines architecturally efficient students with logit-based KD as an evaluated option and reports both FP32 and estimated INT8 footprints as deployment-oriented size indicators. Because no measured post-quantization accuracy is available in the present version, the INT8 values are interpreted strictly as storage estimates rather than as validated quantized-model results.

### 2.4 Edge and mobile AI

Edge-AI frameworks and mobile-oriented backbones such as MobileNetV3 Howard et al. (2019), EfficientNet-Lite Tan and Le (2021), and ShuffleNet Zhang X. et al. (2018) have shown that careful architectural design can significantly reduce latency and memory on resource-constrained devices. Ultra-compact designs such as MCUNet Lin et al. (2020) push this further to microcontroller-class hardware. We work in the same spirit. Rather than co-designing a new architecture, we compare existing lightweight backbones and select LCNet-0.5 because it offers the most favorable balance between size and accuracy under the sub-megabyte constraint. The Raspberry Pi prototype then demonstrates workflow feasibility, while a full inference-only latency and power benchmark remains an open validation step.

## 3 Mathematical framework

### 3.1 Problem formulation

Let $D={\left\{\left(x_{i},y_{i}\right)\right\}}_{i=1}^{N}$ denote the TrashNet dataset with *N* labeled samples, where $x_{i}\in {\mathbb{R}}^{H\times W\times 3}$ is an RGB image and *y*_*i*_∈{1, …, *C*} is the corresponding class label among *C* = 6 waste categories. A deep neural network parameterized by θ is represented as a mapping $f_{\theta }:{\mathbb{R}}^{H\times W\times 3}\to {\mathbb{R}}^{C}$ that produces logits *z*_*i*_ = *f*_θ_(*x*_*i*_).

The teacher *f*_*T*_ (MobileNetV4) has parameters θ_*T*_. The students ${\left\{f_{S}^{\left(k\right)}\right\}}_{k=1}^{K}$, with parameters ${\left\{\theta_{S}^{\left(k\right)}\right\}}_{k=1}^{K}$, correspond to EfficientNet-Lite0, LCNet-0.5, and MobileNetV3-Small-0.5 (*K* = 3). The goal is to train each $f_{S}^{\left(k\right)}$ so that it achieves strong predictive performance on D while remaining substantially smaller and cheaper than *f*_*T*_, and, therefore, suitable for deployment on CPU-only edge devices. The distillation objective is described below for a single generic student *f*_*S*_ and applied independently to each student architecture.

### 3.2 Knowledge distillation objective

Knowledge distillation transfers information from teacher to student through softened output distributions Hinton et al. (2015). Given teacher logits *z*_*T, i*_ = *f*_*T*_(*x*_*i*_) and student logits *z*_*S, i*_ = *f*_*S*_(*x*_*i*_), the softened probabilities at temperature τ>1 are given by Equation 1.


$$\begin{array}{l} p_{T,i}^{\left(\tau \right)}=\text{softmax}\left(\frac{z_{T,i}}{\tau }\right),\;p_{S,i}^{\left(\tau \right)}=\text{softmax}\left(\frac{z_{S,i}}{\tau }\right). \end{array}$$ (1)

The distillation loss is the Kullback–Leibler divergence between these two distributions (Equation 2),


$$\begin{array}{l} L_{\text{KD}}=\frac{\tau^{2}}{N}\overset{N}{\underset{i=1}{\sum }}\text{KL}\left(p_{T,i}^{\left(\tau \right)}||p_{S,i}^{\left(\tau \right)}\right), \end{array}$$ (2)

where the KL term is defined in Equation 3.


$$\begin{array}{l} \text{KL}\left(p_{T,i}^{\left(\tau \right)}||p_{S,i}^{\left(\tau \right)}\right)=\overset{C}{\underset{c=1}{\sum }}p_{T,i,c}^{\left(\tau \right)}\log\frac{p_{T,i,c}^{\left(\tau \right)}}{p_{S,i,c}^{\left(\tau \right)}}. \end{array}$$ (3)

The τ^2^ factor is the standard scaling that keeps gradient magnitudes comparable across temperatures Hinton et al. (2015).

### 3.3 Supervised loss and total objective

The student is also trained on the ground-truth labels using cross-entropy with label smoothing. Let


$$\begin{array}{l} p_{S,i}=\text{softmax}\left(z_{S,i}\right) \end{array}$$ (4)

be the standard, temperature-free predicted probabilities (Equation 4), and let the label-smoothed target distribution $q_{i}\in {\mathbb{R}}^{C}$ defined in Equation 5,


$$\begin{array}{l} q_{i,c}=\{\begin{array}{cc} 1-\varepsilon , & \text{if }c=y_{i},\\ \frac{\varepsilon }{C-1}, & \text{otherwise}, \end{array} \end{array}$$ (5)

with smoothing factor ε = 0.1. The supervised loss is given by Equation 6.


$$\begin{array}{l} L_{\text{CE}}=-\frac{1}{N}\overset{N}{\underset{i=1}{\sum }}\overset{C}{\underset{c=1}{\sum }}q_{i,c}\log p_{S,i,c}. \end{array}$$ (6)

The total objective combines the two terms,


$$\begin{array}{l} L_{\text{total}}=\left(1-\alpha \right)L_{\text{CE}}+\alpha L_{\text{KD}}, \end{array}$$ (7)

where α∈[0, 1] controls how much the student trusts the teacher relative to the ground-truth labels. In the experiments we fix the temperature τ and pick α to favor the teacher's soft targets while keeping a non-negligible hard-label term. Optimization uses AdamW with a cosine-annealed learning rate over *E* training epochs.

### 3.4 Model size estimation

Let *P*(θ) denote the number of trainable parameters of a network and let *b* be the number of bytes per parameter at a chosen precision. The approximate weight-storage footprint is defined in Equation 8.


$$\begin{array}{l} M\left(\theta ,b\right)=\frac{P\left(\theta \right)b}{1024^{2}}\;\text{MB}. \end{array}$$ (8)

We report the FP32 footprint (*b* = 4) and an estimated INT8 footprint (*b* = 1), together with the actual checkpoint file size on disk. The MobileNetV4 teacher has roughly 3.77 M parameters; the three selected students have 3.38 M (EfficientNet-Lite0), 0.61 M (LCNet-0.5), and 0.57 M (MobileNetV3-Small-0.5) parameters. These are substantial reductions in parameter count and footprint for the two smallest students. The extent to which KD closes the accuracy gap is treated as an empirical question and evaluated against no-KD baselines in the cross-validation analysis.

## 4 Methodology

### 4.1 System overview

The goal is to obtain compact image classifiers that are accurate enough for practical waste sorting while fitting within the tight memory budgets of edge devices. The framework has three stages.

First, a high-capacity MobileNetV4 model is trained on TrashNet under standard supervised learning and serves as the reference teacher for KD experiments. Second, three compact student architectures—EfficientNet-Lite0, LCNet-0.5, and MobileNetV3-Small-0.5—are trained, each one independently, with the KD objective in Equation 7 for the main single-split comparison; in the five-fold study, the same students are also trained without KD for a direct ablation. Third, the models are compared on classification accuracy and objective size indicators, and a size–accuracy rule selects the student that best fits a sub-megabyte estimated INT8 budget.

Each stage uses the same input pipeline (224 × 224 resize, ImageNet normalization, and standard augmentation), the same optimizer (AdamW with cosine annealing), and the same batch size. Holding these variables constant reduces training-recipe confounding, although the shorter cross-validation budget means that single-split and cross-validation results are interpreted separately.

### 4.2 Teacher training

The teacher *f*_*T*_ is instantiated as mobilenetv4_conv_small.e1200_r224_in1k from the timm library, initialized with ImageNet-pretrained weights and adapted to six output classes. It is fine-tuned on TrashNet with cross-entropy. Inputs are resized to 224 × 224 and normalized with ImageNet statistics. The augmentation pipeline at training time consists of random resized crops and horizontal flips, and the validation pipeline applies a center crop only.

We train for *E*_*T*_ epochs with AdamW, an initial learning rate of 2 × 10^−4^, weight decay of 0.02, a cosine-annealing schedule, and a batch size of 64. The teacher reaches a top-1 accuracy of 97.09% on the held-out test set, and its parameters are frozen for the distillation stage.

### 4.3 Student distillation

Each student is instantiated from timm and modified to predict six classes. The preprocessing pipeline matches the teacher's, which keeps the input distribution comparable.

KD training follows Equation 7. For each labeled sample (*x*_*i*_, *y*_*i*_), the teacher logits *z*_*T, i*_ and student logits *z*_*S, i*_ are computed; the supervised loss $L_{\text{CE}}$ uses label-smoothed cross-entropy with the hard labels *y*_*i*_, and $L_{\text{KD}}$ uses the KL divergence between the teacher and student softmax outputs at temperature τ. The no-KD baseline sets α = 0 and, therefore, optimizes the supervised label-smoothed cross-entropy only. All students share the same optimizer, schedule, batch size, and epoch budget within each experimental regime. This shared configuration allows architectures and KD/no-KD variants to be compared without changing the data pipeline.

### 4.4 Class imbalance and augmentation protocol

TrashNet is moderately imbalanced, with the *trash* class containing substantially fewer images than the recyclable classes. We, therefore, report macro-averaged metrics in addition to top-1 accuracy and interpret per-class errors using the confusion matrices. This is important because high overall accuracy can hide weak recognition of minority classes, especially when the minority category is visually heterogeneous.

In the present experiments, the baseline training augmentation is deliberately simple—random resized crop and horizontal flip—so that the effect of architecture and distillation is not confounded by heavy augmentation recipes. More advanced sample-mixing methods such as Mixup and CutMix are discussed as direct extensions in Section 9, where they are treated as future controlled ablations rather than as results of the present study.

### 4.5 Model selection criterion

After training, each model is evaluated on the test set and characterized by three quantities: top-1 accuracy, parameter count *P*(θ), and estimated FP32/INT8 size *M*(θ, *b*) defined in Section 3. The teacher provides an upper accuracy bound but a relatively large footprint. Among the students, LCNet-0.5 has the highest top-1 accuracy at a fraction of the teacher's size, EfficientNet-Lite0 is competitive but larger, and MobileNetV3-Small-0.5 is smaller but loses more accuracy.

We adopt a simple size–accuracy rule: among candidates whose estimated INT8 footprint is below 1 MB, pick the model with the highest top-1 accuracy. This rule reflects the deployment constraint that matters most in TinyML scenarios. Under this rule, LCNet-0.5 is selected as the primary candidate for the edge prototype in Section 7.

## 5 Experimental setup

### 5.1 Dataset

We evaluate the framework on the public TrashNet dataset Thung and Yang (2016), which contains 2,527 RGB images across six classes: *cardboard, glass, metal, paper, plastic*, and *trash*. The dataset is moderately imbalanced: *trash* contains only 137 images, while the other classes contain roughly 400–600 each. This imbalance shapes per-class recall in a predictable way and is considered in the per-class analysis.

Within each class, images vary in object shape, scale, packaging, and texture. The acquisition setup is, however, relatively controlled: objects are typically placed on a white posterboard under sunlight and/or ambient room lighting. Backgrounds are simple, but illumination still varies, and shadows are visible. Specular highlights are common for *glass* and *metal*, and some samples are ambiguous (mixed materials), which is the natural source of residual confusion among *glass, metal*, and *plastic*, and between *trash* and visually similar recyclables.

Table 2 reports per-class counts and the stratified 70%/15%/15% split (1, 769/379/379 images) used for model selection in the main experiments. The dataset is used as provided, with no manual relabelling; only file integrity (readable images) is checked. All images are resized to 224 × 224 and normalized with ImageNet statistics, which is the standard operating point for the ImageNet-pretrained backbones used in this study.

**Table 2:** TrashNet class distribution and stratified split statistics (70%/15%/15%).

Class	Total	Train	Val	Test
Cardboard	403	282	60	61
Glass	501	351	75	75
Metal	410	287	62	61
Paper	594	416	89	89
Plastic	482	337	72	73
Trash	137	96	21	20
Total	2,527	1,769	379	379

### 5.2 Training configuration

All models—teacher and students—share the same optimization settings unless noted otherwise. We use AdamW with an initial learning rate of 2 × 10^−4^, weight decay of 0.02, and cosine-annealing of the learning rate over *E* epochs. The batch size is fixed at 64. Mixed-precision (FP16) training is enabled where available to reduce memory pressure, but all reported model sizes are computed assuming full-precision FP32 or INT8 weights.

The teacher is trained with standard cross-entropy. In the main single-split experiment, each student is trained with the combined KD objective using temperature τ = 4 and mixing coefficient α = 0.8. These values are common logit-distillation defaults, but they are not claimed to be optimal because a full τ–α sweep was not run. In the cross-validation experiment, each student is evaluated both with KD and without KD; the no-KD variant corresponds to α = 0 and uses the same supervised objective and augmentation pipeline.

### 5.3 Evaluation protocol

The main accuracy and size comparison is reported on the single stratified 70%/15%/15% split described above, with the best checkpoint selected by validation accuracy and evaluated once on the held-out test set. This main split uses the longer 100-epoch training budget in Table 3. To strengthen the statistical reliability of the comparison, we additionally re-train every model under a stratified five-fold cross-validation protocol. The dataset is partitioned into five-folds with class proportions preserved; for each fold, the teacher and the three students are re-trained from scratch, and the held-out fold is used for evaluation. The five-fold run uses a shorter budget (*E*_*T*_ = 40, *E*_*S*_ = 50) to make the repeated training feasible. Therefore, the CV values are used to assess ranking stability and KD/no-KD behavior, not as direct numerical replacements for the 100-epoch single-split results. We report mean and standard deviation of top-1 accuracy and macro-F1 across the five-folds, together with 95% confidence intervals computed from the *t*-distribution.

**Table 3:** Main hyperparameters for the single-split and cross-validation regimes.

Hyperparameter	Value	Notes / rationale
Number of classes (*C*)	6	TrashNet classes
Input resolution	224 × 224	Standard for ImageNet-pretrained backbones in timm
Batch size	64	Fixed for teacher and students
Optimizer	AdamW	Used for all models
Learning rate	2 × 10^−4^	Base learning rate
Weight decay	0.02	AdamW regularization
LR schedule	Cosine annealing	*T*_max_ matched to each regime
Teacher epochs (*E*_*T*_), main split	100	Supervised training on the 70/15/15 split
Student epochs (*E*_*S*_), main split	100	KD training for each student on the 70/15/15 split
Teacher epochs (*E*_*T*_), five-fold CV	40	Shorter cross-validation budget
Student epochs (*E*_*S*_), five-fold CV	50	KD and no-KD variants under the same fold budget
Label smoothing (ε)	0.1	Applied in cross-entropy
KD temperature (τ)	4.0	Fixed default; not tuned in a full sweep
KD mixing (α)	0.8	Fixed default for KD variants; no-KD sets α = 0

For each test image, predictions are produced by single-crop inference: the image is resized and center-cropped to 224 × 224, normalized, and passed through the network. The output logits are converted to class probabilities by softmax, and the predicted label is the top-1 class. Each test image is evaluated exactly once.

### 5.4 Evaluation metrics

We use three primary metrics: top-1 accuracy (%) on the held-out test set, parameter count (in millions), and model size (MB), estimated from the parameter count for FP32 and INT8 precisions and also reported as the actual on-disk checkpoint size. For the selected LCNet-0.5 student, we additionally report macro-averaged precision, recall, and F1 computed from the test-set confusion matrix. The hardware section reports the observed end-to-end camera-to-display latency of the Raspberry Pi prototype workflow.

### 5.5 Experimental process and implementation details

The experimental process follows a fixed pipeline designed to ensure clear comparisons between architectures and training variants. First, TrashNet is split using class-stratified sampling into the main training, validation, and test partitions reported in Table 2. All images are resized or cropped to 224 × 224 and normalized using ImageNet statistics, ensuring that the teacher and all student models receive inputs from the same preprocessing pipeline. The MobileNetV4 teacher is trained on the main training split, selected according to validation accuracy, evaluated once on the held-out test set, and then frozen for the student-training experiments.

Second, the three compact student models are trained using the same preprocessing, optimizer, learning-rate schedule, batch size, and augmentation settings listed in Table 3. The main single-split experiment uses KD-trained students for the accuracy–size comparison, whereas the cross-validation experiment trains both KD and no-KD variants of each student under the same fold budget. This design separates the architecture-selection question from the KD-ablation question: the main split identifies the best compact candidate under the size–accuracy rule, while the five-fold study evaluates whether KD improves macro-F1 relative to supervised training alone.

Third, the selected LCNet-0.5 model is evaluated beyond the main TrashNet split. Cross-dataset transfer is assessed on the six RealWaste classes that overlap with TrashNet, first without fine-tuning and then after a short adaptation stage. Finally, the selected student model is exported to ONNX and integrated into the Raspberry Pi workflow. The reported hardware value corresponds to full camera-to-display latency, including image capture, preprocessing, local inference, softmax decoding, and LCD update.

All experiments were implemented in Python using PyTorch, timm, scikit-learn, NumPy, and ONNX Runtime. The main split and five-fold partitions were generated by stratified sampling with fixed random seeds. Unless otherwise stated, the random seed was fixed to 42 for data splitting and model training.

## 6 Results and analysis

This section is organized so that the most direct accuracy–size comparison comes first (Section 6.1), followed by the cross-validated reading (Section 6.2), the per-class diagnostics (Section 6.3), the cross-dataset generalization results (Section 6.4), and qualitative examples (Section 6.5). The physical prototype and its observed end-to-end latency are then described in Section 7.

### 6.1 Main quantitative results

Table 4 summarizes the head-to-head comparison between the MobileNetV4 teacher and the three KD-trained students on the main 100-epoch stratified split. The four models occupy different points on the accuracy–size curve. The teacher reaches 97.09% top-1 accuracy with a 14.4 MB FP32 footprint; EfficientNet-Lite0 reaches 93.99% with a comparable size; LCNet-0.5 reaches 94.18% at only 0.61 M parameters and an estimated 0.58 MB of INT8 weights; MobileNetV3-Small-0.5 lands at 87.73% with a similar size to LCNet-0.5 but with a clearly larger accuracy gap.

**Table 4:** Teacher and student accuracy–size summary on the TrashNet test set.

Model	Arch (timm)	Top-1	Params	FP32 MB	INT8 MB	Ckpt MB
Teacher	mobilenetv4	97.09%	3.77	14.38	3.60	14.82
EffNet-Lite0	efficientnet_lite0	93.99%	3.38	12.89	3.22	13.16
LCNet-0.5	lcnet_050	94.18%	0.61	2.32	0.58	2.40
MobileNetV3-Small-0.5	mobilenetv3_small_050	87.73%	0.57	2.19	0.55	2.30

FP32 and INT8 MB are storage estimates derived from the parameter count (four and one bytes per parameter, respectively); “Ckpt MB” is the actual on-disk checkpoint size.

Two observations matter for deployment. First, model size follows directly from parameter count: assuming FP32, each parameter takes 4 bytes; with INT8 storage, this drops to 1 byte. The teacher and EfficientNet-Lite0 remain above ~3 MB even in estimated INT8 form, while LCNet-0.5 and MobileNetV3-Small-0.5 fall well-below 1 MB. These INT8 values are storage estimates only; they are not a substitute for measured quantized-model accuracy. Second, LCNet-0.5 is the model that pays the smallest accuracy price for a given size reduction on the main split: relative to the teacher it gives up about 2.91% points of accuracy (94.18% vs. 97.09%) in exchange for a more than 6 × reduction in parameter count and several megabytes less storage. The supported conclusion is, therefore, an architecture-selection conclusion: LCNet-0.5 is the most attractive sub-megabyte candidate among the evaluated models.

### 6.2 Five-fold cross-validation

To complement the single-split numbers, Table 5 reports mean ± std and 95% confidence intervals across five stratified folds for top-1 accuracy and macro-F1. The cross-validation run was executed with *N*_folds_ = 5, *E*_*T*_ = 40 teacher epochs, and *E*_*S*_ = 50 student epochs. These settings are shorter than the main single-split training budget (100 epochs), which partly explains why the cross-validated teacher mean is much lower than the main-split teacher accuracy. Consequently, the two regimes are not presented as directly interchangeable. The single split characterizes the best 100-epoch model used for the primary size–accuracy comparison; the CV run characterizes fold stability and the KD/no-KD ablation under a consistent repeated-training budget.

**Table 5:** Stratified five-fold cross-validation on TrashNet.

Model	Top-1 (%)	95% CI (%)	Macro-F1	95% CI
EfficientNet-Lite0 (no KD)	91.93 ± 1.02	[90.67, 93.19]	0.9030 ± 0.0149	[0.8845, 0.9216]
EfficientNet-Lite0 (KD)	91.73 ± 1.25	[90.18, 93.28]	0.9008 ± 0.0128	[0.8849, 0.9167]
Teacher (MobileNetV4)	89.75 ± 1.79	[87.54, 91.97]	0.8797 ± 0.0202	[0.8547, 0.9047]
LCNet-0.5 (KD)	87.65 ± 1.64	[85.62, 89.68]	0.8564 ± 0.0162	[0.8364, 0.8765]
LCNet-0.5 (no KD)	87.58 ± 2.58	[84.37, 90.78]	0.8586 ± 0.0275	[0.8245, 0.8927]
MobileNetV3-Small-0.5 (no KD)	81.01 ± 2.59	[77.80, 84.22]	0.7895 ± 0.0227	[0.7613, 0.8177]
MobileNetV3-Small-0.5 (KD)	78.59 ± 2.99	[74.88, 82.31]	0.7640 ± 0.0296	[0.7272, 0.8008]

Mean ± std across folds and 95% confidence intervals computed with the *t*-distribution (*n*= 5). Student rows include both KD-trained and no-KD variants where available.

For completeness, Table 6 gives the fold-level scores for the teacher and for each student with and without distillation. The fold-wise view is useful because the minority *trash* class is small; a few samples can, therefore, noticeably affect per-class recall and macro-F1.

**Table 6:** Per-fold final scores in the five-fold cross-validation experiment.

Fold	Model	KD setting	Top-1	Macro-F1	Trash recall
1	Teacher	–	0.8854	0.8663	–
1	EffLite0	With KD	0.9051	0.8884	0.9286
1	EffLite0	No KD	0.9111	0.8982	1.0000
1	LCNet-0.5	With KD	0.8498	0.8298	0.9286
1	LCNet-0.5	No KD	0.8735	0.8581	1.0000
1	MNv3-S-0.5	With KD	0.7391	0.7187	0.9286
1	MNv3-S-0.5	No KD	0.7826	0.7685	1.0000
2	Teacher	–	0.8854	0.8695	–
2	EffLite0	With KD	0.9032	0.8865	0.8571
2	EffLite0	No KD	0.9170	0.9004	0.8571
2	LCNet-0.5	With KD	0.8735	0.8534	0.8929
2	LCNet-0.5	No KD	0.8498	0.8358	0.9286
2	MNv3-S-0.5	With KD	0.7747	0.7532	0.9286
2	MNv3-S-0.5	No KD	0.7846	0.7652	0.9286
3	Teacher	–	0.9208	0.9072	–
3	EffLite0	With KD	0.9287	0.9053	0.8519
3	EffLite0	No KD	0.9188	0.8951	0.7407
3	LCNet-0.5	With KD	0.8871	0.8660	0.8889
3	LCNet-0.5	No KD	0.8990	0.8844	0.8519
3	MNv3-S-0.5	With KD	0.7980	0.7692	0.7407
3	MNv3-S-0.5	No KD	0.8257	0.7968	0.7778
4	Teacher	–	0.8832	0.8608	–
4	EffLite0	With KD	0.9287	0.9157	0.9630
4	EffLite0	No KD	0.9366	0.9292	1.0000
4	LCNet-0.5	With KD	0.8911	0.8706	0.9630
4	LCNet-0.5	No KD	0.9050	0.8877	1.0000
4	MNv3-S-0.5	With KD	0.8139	0.7930	0.9630
4	MNv3-S-0.5	No KD	0.8158	0.7971	0.9630
5	Teacher	–	0.9129	0.8948	–
5	EffLite0	With KD	0.9208	0.9081	0.9259
5	EffLite0	No KD	0.9129	0.8923	0.8148
5	LCNet-0.5	With KD	0.8812	0.8624	0.8519
5	LCNet-0.5	No KD	0.8515	0.8272	0.8148
5	MNv3-S-0.5	With KD	0.8040	0.7858	1.0000
5	MNv3-S-0.5	No KD	0.8416	0.8198	0.8889

Trash recall is reported only for student models because it was not logged for the teacher.

Table 7 compares each KD-trained student against the same architecture trained without distillation using the paired Wilcoxon-test across folds. The test was set up as a one-sided comparison in favor of KD (with-KD > no-KD). The observed differences are small and not statistically significant at conventional thresholds; in this particular run, no-KD slightly exceeds with-KD in mean macro-F1 for all three students. We, therefore, interpret the five-fold experiment conservatively: KD is competitive under this short cross-validation budget, but the evidence does not support a statistically significant KD advantage over the no-KD baseline.

**Table 7:** Paired Wilcoxon significance test comparing with-KD against no-KD macro-F1 across the five-folds.

Student	no-KD mF1	with-KD mF1	Δ mF1	Wilcoxon stat.	*p*-value
EfficientNet-Lite0	0.9030	0.9008	-0.0023	7.0	0.5938
LCNet-0.5	0.8586	0.8564	-0.0022	7.0	0.5938
MobileNetV3-Small-0.5	0.7895	0.7640	-0.0255	0.0	1.0000

The alternative hypothesis is with-KD > no-KD.

The cross-validated ranking is broadly consistent with the single-split accuracy–size picture: EfficientNet-Lite0 gives the strongest accuracy and macro-F1, with its KD and no-KD variants nearly tied, while LCNet-0.5 remains the most attractive sub-megabyte candidate under the size–accuracy rule. The Wilcoxon comparison also clarifies that the main deployment conclusion should not rest only on KD improving every student; it rests on the combination of compact architecture, competitive accuracy, and the sub-megabyte INT8 footprint of LCNet-0.5.

### 6.3 Per-class analysis

**Figure 1:**
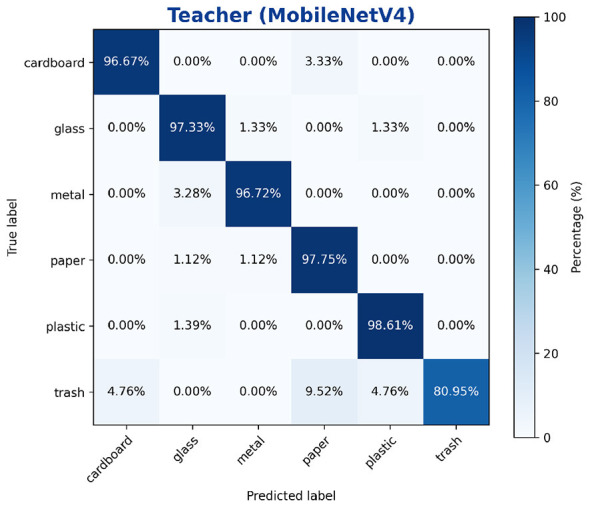
Confusion matrix of the MobileNetV4 teacher on the TrashNet test set. Strong diagonal dominance, consistent with top-1 accuracy 97.09% and macro-averaged precision, recall, and F1 of 0.9750, 0.9512, and 0.9613. Residual errors concentrate in visually similar categories, primarily *trash* predicted as *paper* or *plastic*, with occasional confusion among *glass, metal*, and *plastic*.

**Figure 2:**
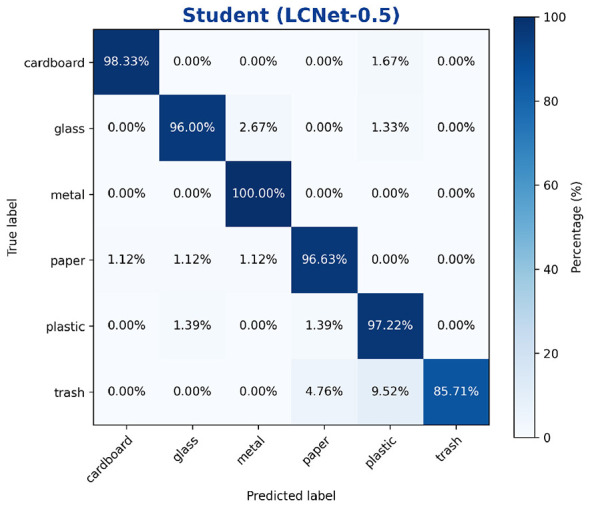
Confusion matrix of the LCNet-0.5 student on the TrashNet test set. Top-1 accuracy 94.18%; macro-averaged precision, recall, and F1 of 0.9529, 0.9083, and 0.9247. The main degradation appears in the *trash* class, which is sometimes predicted as *cardboard, paper, metal*, or *plastic*. Confusion among *glass, metal*, and *plastic* is mild.

For the selected LCNet-0.5 student, macro-averaged precision, recall, and F1 on the single test split are 0.9529, 0.9083, and 0.9247. The teacher reaches 0.9750, 0.9512, and 0.9613 on the same split. The gap between teacher and student is, therefore, visible primarily in recall, and the confusion matrices in Figures 1, 2 explain why: both matrices are strongly diagonally dominant, but the student's main degradation is concentrated in the under-represented *trash* class, where samples are most likely to be misread as visually similar recyclables (*cardboard, paper, metal*, or *plastic*). The recyclable classes themselves remain well-separated.

**Figure 3:**
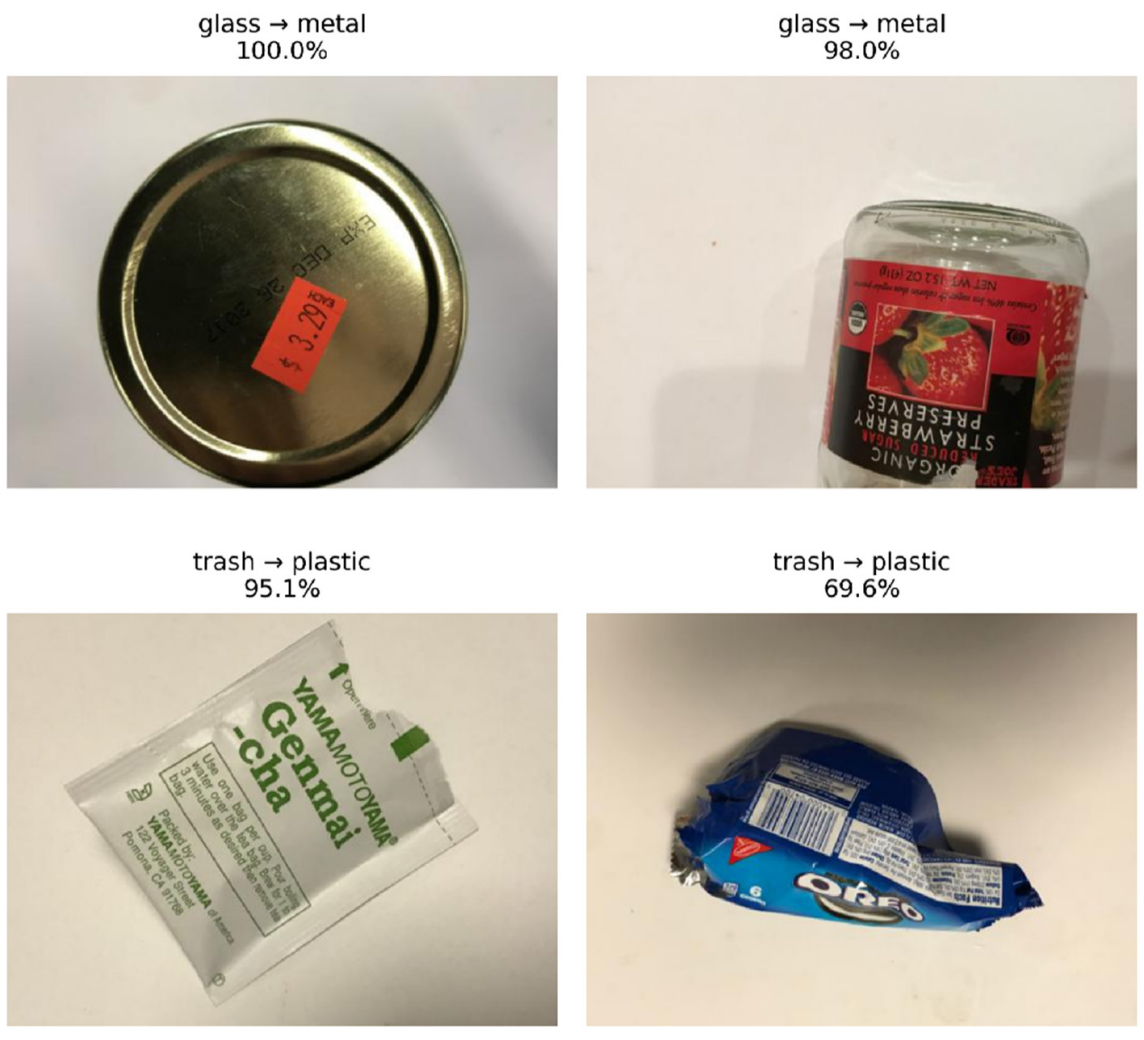
Representative misclassified TrashNet samples for the LCNet-0.5 student. Most errors occur on visually ambiguous or low-quality images, where class-specific cues are weak. Images from “Dataset for Image-Based Waste Classification” by Gary Thung, licensed under MIT License.

A closer look at the misclassified samples (Figure 3) shows that residual errors are mostly visually ambiguous cases: low-quality images, mixed-material items, or objects whose dominant cue overlaps two classes. This shape of error matters: it suggests that the remaining headroom is not primarily a model-capacity issue but is also tied to dataset size, class balance, and visual ambiguity.

### 6.4 Cross-dataset generalization

To understand how well the compressed model transfers beyond TrashNet, we evaluate the LCNet-0.5 student on the public RealWaste dataset Single et al. (2023), restricted to the six categories that overlap with TrashNet (*cardboard, glass, metal, paper, plastic*, and *trash*). RealWaste is randomly split into train/validation/test partitions per class (for example, 322/69/70 for cardboard and 644/138/139 for plastic), yielding a held-out test set of 541 images.

Evaluated zero-shot, the LCNet-0.5 student (trained only on TrashNet) reaches 41.04% top-1 accuracy and macro-F1 0.3648 on RealWaste, despite reaching 94.18% on the TrashNet test set. The gap is large, but it is consistent with the appearance differences between the two datasets: TrashNet uses a controlled white-posterboard background, while RealWaste contains realistic landfill imagery with cluttered backgrounds and partial occlusions. We then fine-tune the student on RealWaste with a two-stage schedule: a 3-epoch classifier-head warm-up, followed by full-network fine-tuning. The best validation accuracy reaches 82.31%, and the test-set accuracy reaches 79.67% with macro-F1 0.7929. Per-class F1 is now balanced (for example, 0.8376 for glass and 0.8333 for metal). The price paid for this specialization is reduced in-domain performance on TrashNet, which drops to 65.08% accuracy and macro-F1 0.5739—the expected trade-off of cross-domain adaptation without retention mechanisms.

These results give a fair reading of where the selected compact student stands today. Strict zero-shot generalization is limited; with a small fine-tuning step, the same student adapts comfortably to substantially different real-world imagery. This is consistent with the edge-deployment scenario we target, where a small calibration/fine-tuning step on locally collected images is realistic.

### 6.5 Qualitative analysis

**Figure 4:**
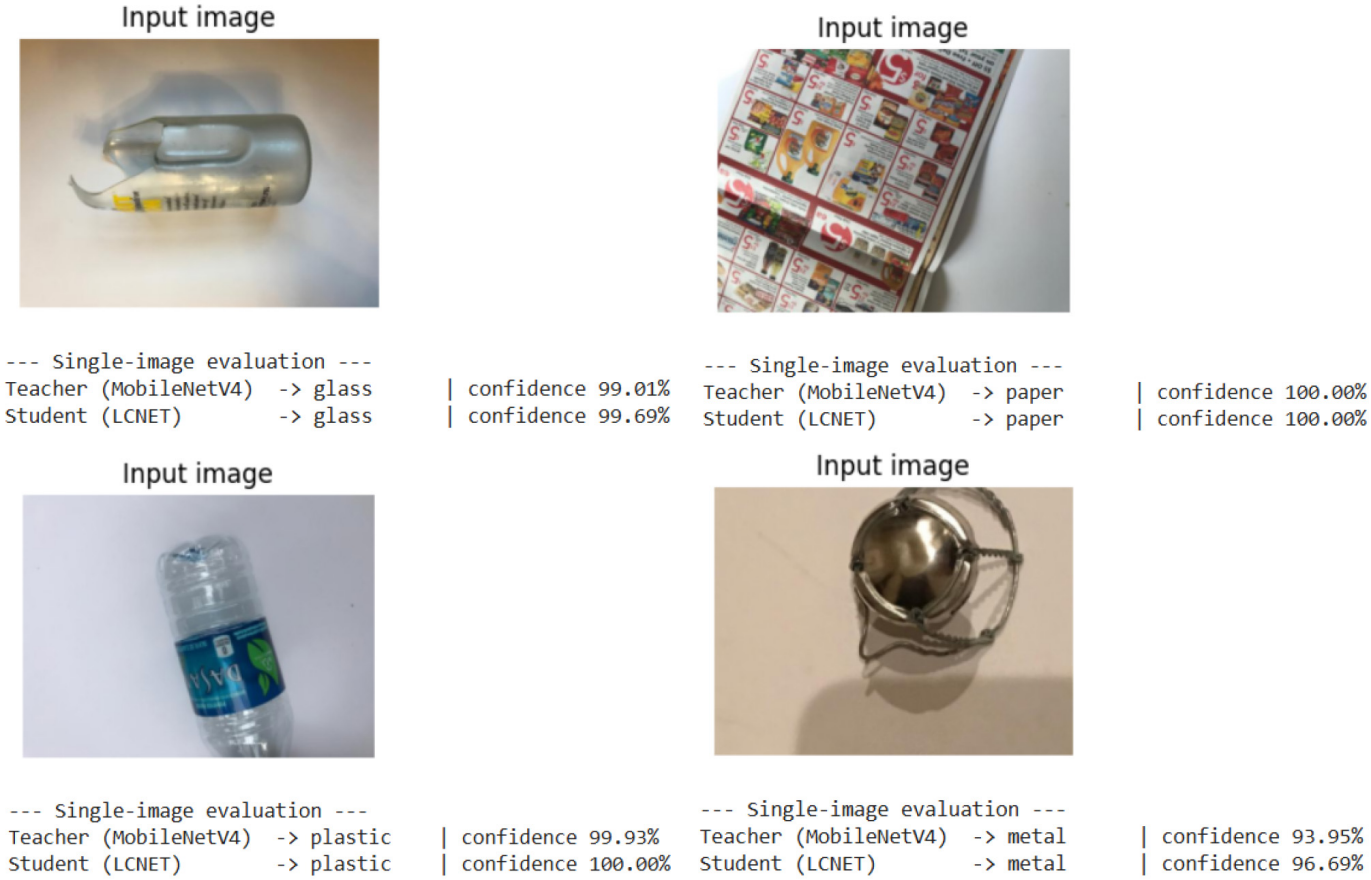
Qualitative examples from the TrashNet test set. The MobileNetV4 teacher and all three selected students predict the correct class on each sample (labels omitted for brevity); the compressed models closely match the teacher's behavior on typical test images. Images from “Dataset for Image-Based Waste Classification” by Gary Thung, licensed under MIT License.

Figure 4 shows representative test-set examples. For these images, the MobileNetV4 teacher and all three compact students predict the correct class. The agreement is encouraging: it indicates that, on typical TrashNet images, the compact models match the teacher's decisions and not only its summary accuracy.

**Figure 5:**
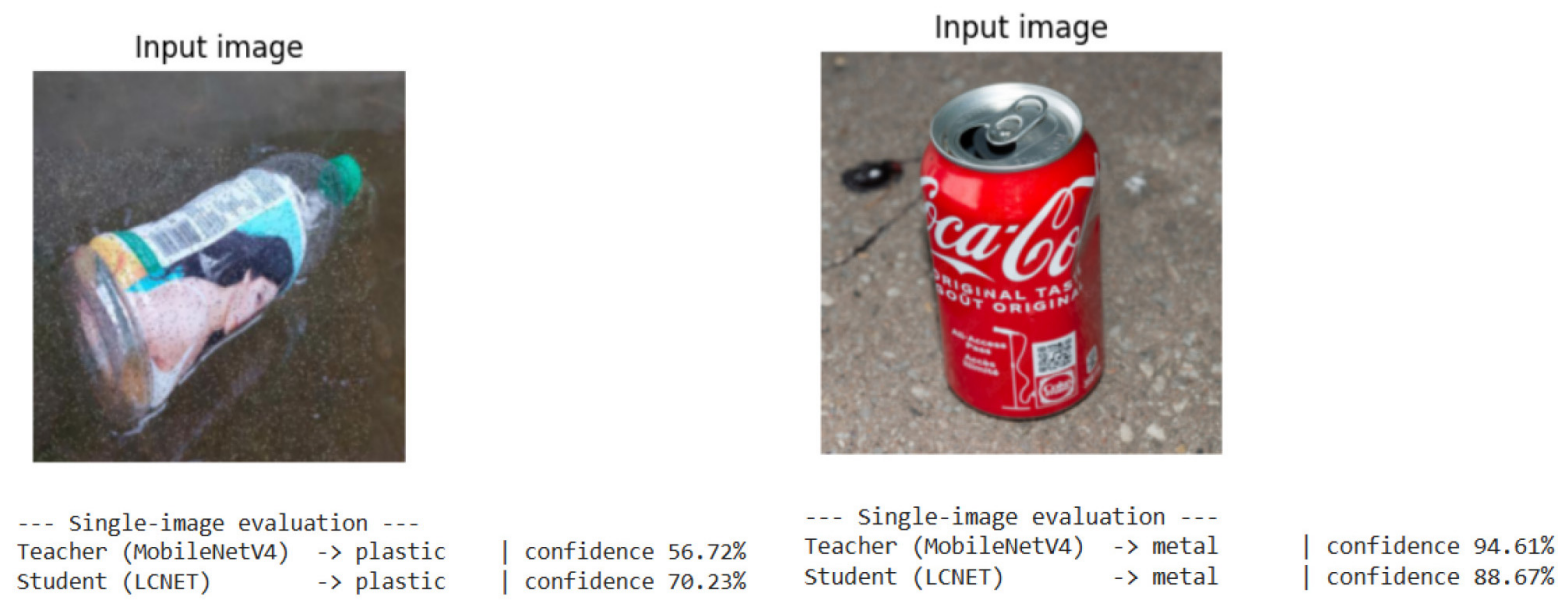
Qualitative examples from unseen images not part of the TrashNet dataset. The LCNet-0.5 student produces plausible predictions despite variations in lighting, background, and viewpoint, suggesting reasonable robustness beyond the training distribution.

To probe robustness beyond TrashNet, LCNet-0.5 is additionally evaluated on a small collection of uncurated web images that include variations in lighting, background clutter, and viewpoint (Figure 5). The student remains stable on these inputs, with most observed errors occurring in visually similar pairs (paper vs. cardboard, plastic vs. general trash). These examples are not a replacement for the RealWaste test above, but they provide a visual sanity check that the model is not limited to the most canonical TrashNet views.

## 7 Hardware prototype and edge deployment workflow

The accuracy and size results above characterize the selected LCNet-0.5 student in offline conditions. To demonstrate practical feasibility, the selected student is integrated into a self-contained Raspberry Pi prototype that performs all inference locally. The prototype shows how the compact model fits into a complete camera-to-feedback loop on a low-cost, CPU-only single-board computer without cloud inference.

### 7.1 System architecture

**Figure 6:**
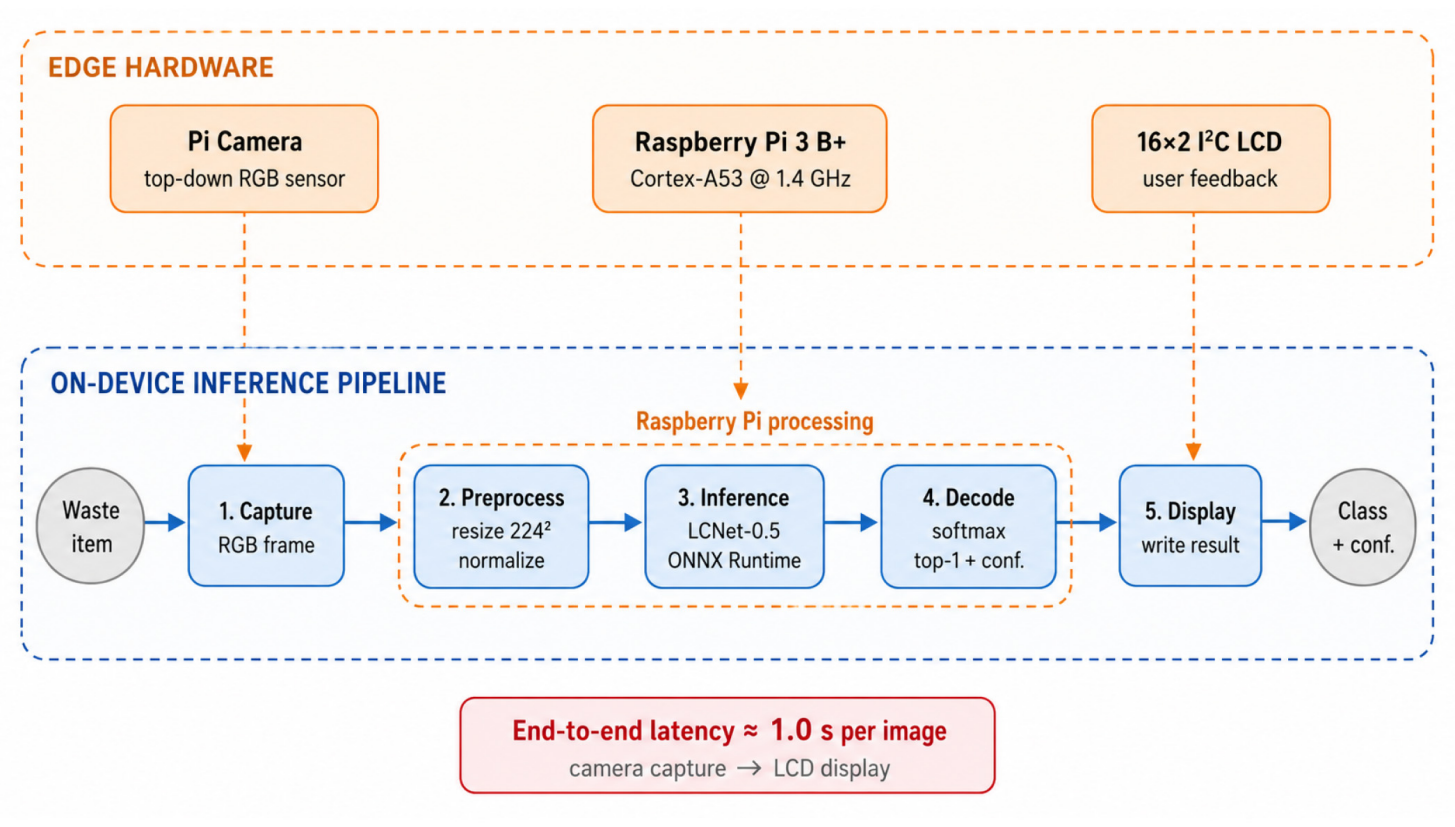
Edge deployment workflow of the LCNet-0.5 student on the Raspberry Pi 3 Model B+ prototype. The top lane shows the physical hardware components, while the bottom lane shows the five-step on-device inference pipeline. Solid blue arrows indicate the left-to-right data flow from the waste item to the displayed class label. Dashed orange arrows indicate the hardware component responsible for each part of the pipeline: the Pi Camera captures the image, the Raspberry Pi performs preprocessing, inference, and decoding, and the LCD displays the predicted class and confidence. The observed end-to-end latency is approximately 1.0 s per image for the full camera-to-display loop.

**Figure 7:**
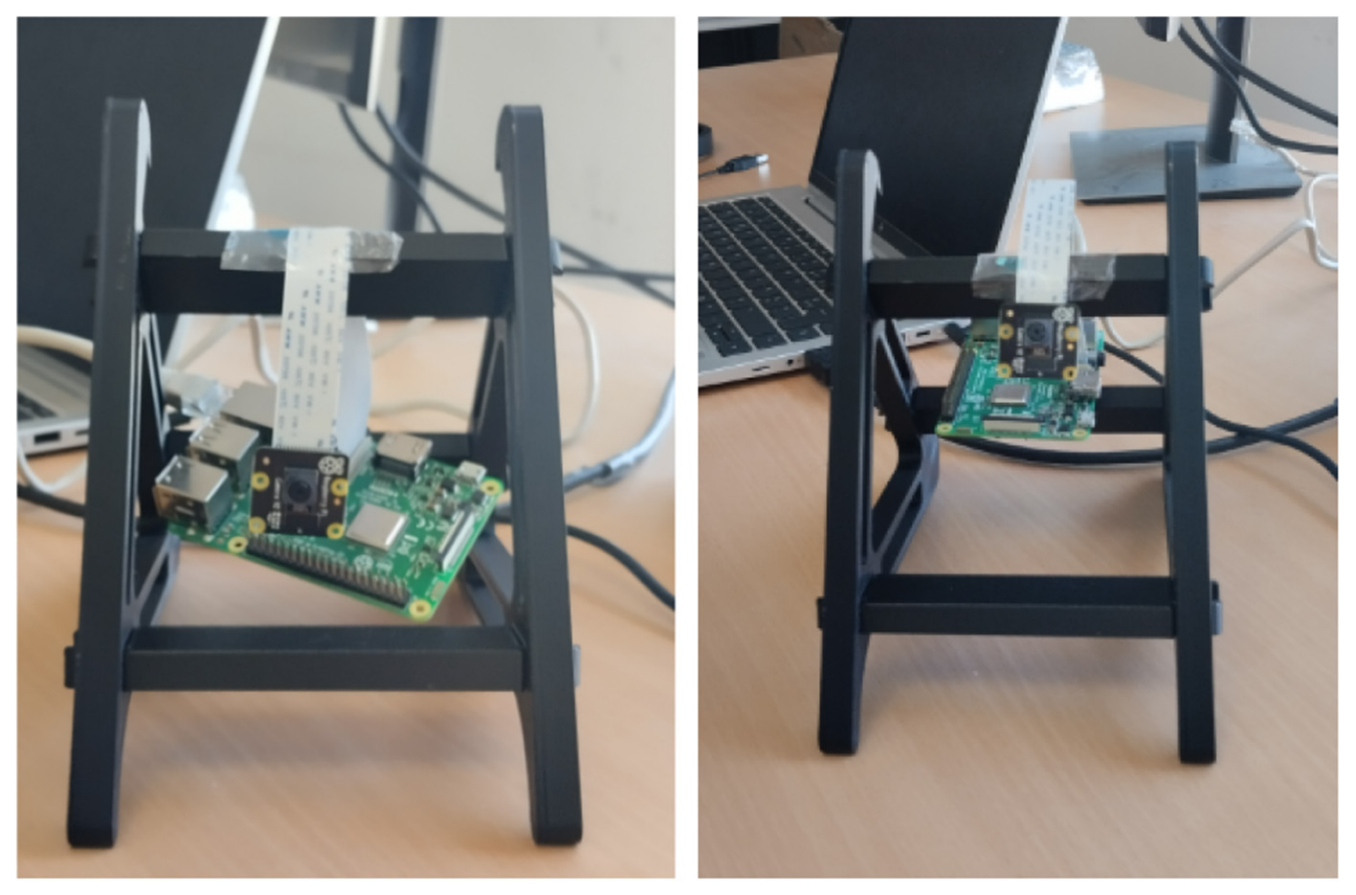
Assembled prototype bench used to integrate the selected LCNet-0.5 student into a camera-based waste-sorting workflow. The Raspberry Pi 3 Model B+ performs all inference locally; the LCD provides immediate user feedback (predicted class and confidence).

Figure 6 summarizes the prototype in a single view: the top row lists the four physical components, the bottom row shows the on-device inference pipeline, and dashed connectors indicate which component implements each pipeline stage. Photographs of the assembled bench are shown in Figure 7.

The four hardware blocks shown in Figure 6 are:

- **Compute platform**. A Raspberry Pi 3 Model B+ (Broadcom BCM2837B0, quad-core ARM Cortex-A53 @ 1.4 GHz, 1 GB LPDDR2 RAM) acts as the edge node. The Raspberry Pi 3 is intentionally chosen as a conservative, CPU-only baseline that is representative of low-cost deployments.
- **Imaging sensor**. A Raspberry Pi Camera Module mounted at the apex of the enclosure provides a top-down view of the bin interior. The fixed mounting standardizes framing, perspective, and working distance, keeping the deployment-time input distribution close to the training-time distribution.
- **Illumination**. A diffused LED ring around the lens delivers near-uniform top-down lighting, which reduces specular reflection on metal and glass items, mitigates harsh shadows, and stabilizes color cues—important factors for compact students that have less representational headroom than the teacher.
- **User interface**. A 16 × 2 I^2^C LCD module displays the predicted class and its softmax probability immediately after each inference.

A custom enclosure rigidly fixes the camera, ring light, processing board, and display, so the geometry between sensor and target object is constant during inference.

### 7.2 On-device inference pipeline

The selected LCNet-0.5 model is converted into a deployment-ready format and then executed locally:

1. **Export**. The PyTorch checkpoint is exported to ONNX (opset 17), preserving the trained weights and the six-way classifier head.
2. **Runtime**. On the Raspberry Pi 3, the exported model is executed through ONNX Runtime using the CPU execution provider; no GPU, NPU, or accelerator is used.
3. **Inference loop**. For each captured frame, the bench executes: capture → resize to 224 × 224 and ImageNet normalization → ONNX inference → softmax → top-1 class and confidence → LCD update.

This loop matches the bottom row of Figure 6 stage-for-stage and is also the loop that is timed in Section 7.3.

### 7.3 Prototype latency observation

Table 8 reports the observed end-to-end latency for the selected LCNet-0.5 student on the Raspberry Pi 3 Model B+ prototype. The reported value corresponds to the full user-facing loop in Figure 6: it includes image capture, preprocessing, model inference, softmax, and LCD update. It is, therefore, a conservative, workflow-level measurement rather than an isolated model-kernel benchmark, and it is reported as such throughout the paper.

**Table 8:** Observed prototype latency for the selected LCNet-0.5 student on the Raspberry Pi 3 Model B+ workflow.

Model	Device	Precision/ runtime	End-to-end latency
LCNet-0.5	Raspberry Pi 3 Model B+	FP32 ONNX, CPU EP	≈1.0 s/image

The reported value is end-to-end camera-to-display latency, not isolated model-kernel latency.

### 7.4 User interaction workflow

The bench operates as a single closed loop. A frame is captured by the top-down camera and preprocessed; the LCNet-0.5 student is invoked locally; and the LCD displays the top-1 class together with its softmax probability. With an end-to-end latency of approximately 1.0 s per image, the feedback is fast enough for an interactive educational or household smart-bin prototype, although further runtime optimization would be needed for high-throughput industrial sorting lines. From the user's perspective, the interaction is intentionally minimal: model complexity is hidden behind a direct feedback signal, which keeps the unit easy to operate and helps reinforce correct sorting behavior.

### 7.5 Design principles

Three principles guide the prototype and generalize to other edge-AI deployments of compact vision models:

1. **Edge-centricity**. All inference happens locally on the Raspberry Pi 3. This removes network dependence, eliminates round-trip latency, preserves user privacy (no images leave the device), and supports operation in areas with weak or no connectivity.
2. **Controlled inference environment**. The fixed sensor geometry and diffused illumination standardize the input distribution and narrow the gap between training-time and deployment-time statistics. This is particularly important for compact students, which have less representational headroom to absorb illumination, scale, or background variation.
3. **Human-in-the-loop feedback**. The LCD turns the unit from a passive classifier into an interactive sorting aid: by surfacing the predicted class and its confidence in real time, the system both verifies correct disposal and reinforces correct sorting behavior, aligned with the broader sustainability goal motivating this work.

### 7.6 Scope and limitations of the hardware evaluation

To prevent over-interpretation, the scope of the present hardware evaluation is stated explicitly. First, the reported ≈1.0 s/image figure is a workflow-level observation that includes camera acquisition, host-side preprocessing, ONNX Runtime inference, softmax, and LCD I/O; it is, therefore, an upper bound on the isolated neural-network inference time and should not be compared directly against kernel-only inference numbers reported elsewhere. Second, the present revision does not include instrumented active-power or energy-per-inference measurements: no calibrated USB power meter or shunt-based logger was available on the bench at the time of submission, and we deliberately avoid estimating energy from nominal board power because such an estimate would not be a true measurement. Third, the prototype currently runs an FP32 ONNX graph; the INT8 footprints in Section 6.1 are storage estimates and are not validated by a post-quantized on-device run. Fourth, the comparison is limited to a single device class (Raspberry Pi 3 Model B+) and does not yet cover Jetson-class or microcontroller-class targets, which would shift the operating point along both the latency and the energy axes. A full instrumented benchmark across these axes is the natural next step.

## 8 Discussion

The results support a more conservative message than a simple “KD improves compact waste classification” claim. A MobileNetV4 teacher provides a strong reference point on the main split, and the three compact students occupy distinct points on the accuracy–efficiency curve. However, the five-fold KD/no-KD comparison does not show a statistically significant macro-F1 advantage for KD. In fact, the no-KD variants slightly outperform the KD variants in mean macro-F1 for all three student architectures. The central supported finding is, therefore, not that KD is the source of improvement, but that compact architecture selection matters: LCNet-0.5 offers the best balance among the sub-megabyte candidates under the selected size–accuracy rule.

This reframing resolves the main tension between the single-split and cross-validation experiments. The main split uses a longer 100-epoch budget and reports the strongest held-out performance of the teacher and KD-trained students. The cross-validation run uses shorter teacher and student budgets (40 and 50 epochs) because every model must be retrained five times and, for students, with and without KD. The resulting CV teacher mean of 89.75% should, therefore, not be read as contradicting the single-split teacher accuracy of 97.09%; it reflects a different, shorter repeated-training regime. What the CV experiment does support is the relative stability of the architecture ranking and the absence of a proven KD advantage under the tested budget.

The per-class results show that the remaining errors are concentrated in visually ambiguous examples and in the under-represented *trash* class. This is consistent with the structure of TrashNet: the recyclable classes contain more examples and often have clearer material cues, while the *trash* category is smaller and visually heterogeneous. The cross-dataset evaluation on RealWaste reinforces the same point. Zero-shot transfer from TrashNet to real landfill imagery is limited, but a short fine-tuning stage substantially improves RealWaste performance, which indicates that the selected compact student can adapt to a new deployment domain when a small amount of local data is available.

The Raspberry Pi prototype should be interpreted as a deployment workflow demonstration with a conservative end-to-end latency observation. The selected LCNet-0.5 student runs locally in a complete camera-to-feedback loop at approximately 1.0 s per image, which is adequate for an interactive educational or household smart-bin prototype. This measurement does not replace a full hardware benchmark: isolated inference latency, model load time, throughput, peak memory, active power, and energy per inference still require instrumented measurement under a fixed benchmarking protocol. For the same reason, we do not compare power or energy against baseline models in this version.

The study also clarifies which claims require further ablation. A complete KD study should re-run no-KD baselines under the same 100-epoch budget as the main split and should sweep the temperature τ and mixing coefficient α, because τ = 4 and α = 0.8 may not be optimal. Similarly, the present augmentation pipeline uses random cropping and horizontal flipping only. Mixup and CutMix are important next comparisons, especially for the minority *trash* class, but they are not reported as results here. Finally, the INT8 values in this paper are storage estimates; actual post-training quantization and quantization-aware training must be evaluated before making claims about quantized accuracy or integer-runtime speed.

A small number of additional ablations would further sharpen the picture but were out of scope for the present revision. A full τ–α grid sweep would require re-training each of the three students for every combination on the 100-epoch schedule, multiplying the existing training budget by the size of the grid; given that the current five-fold KD/no-KD comparison already shows no statistically significant KD advantage (Table 7), we judge that this expansion belongs to a dedicated KD-sensitivity study rather than to the present architecture-selection paper. Mixup and CutMix were similarly deferred: introducing them in addition to KD changes two factors at once and would confound the architecture comparison, so they are listed in Section 9 as the next controlled ablation. quantization-aware training (QAT) was not run for the same reason: the present paper reports INT8 footprints as storage estimates, and a QAT study should report measured post-quantized accuracy on the device, which in turn requires the instrumented hardware benchmark described in Section 7.6.

Overall, the paper should be read as a compact-model selection and deployment-readiness study. Its strongest contribution is a transparent comparison of size, accuracy, fold stability, domain shift, and prototype integration for several mobile CNN students. KD remains part of the training framework, but the present evidence does not prove that it improves compact waste classification under the tested settings.

## 9 Conclusions and future work

We presented a compact CNN study for edge-oriented waste classification using a MobileNetV4 reference model and three mobile-friendly students: EfficientNet-Lite0, LCNet-0.5, and MobileNetV3-Small-0.5. On the main 100-epoch TrashNet split, the teacher reaches 97.09% top-1 accuracy, while the compact students provide different accuracy–size trade-offs. LCNet-0.5 is the most attractive memory-constrained candidate because it reaches 94.18% with 0.61 M parameters and an estimated INT8 footprint of roughly 0.58 MB.

The cross-validation analysis requires a conservative interpretation of KD. Under the five-fold budget used here, KD is competitive but not statistically superior to no-KD training in macro-F1. The paired Wilcoxon tests do not support a significant KD advantage, and the no-KD variants slightly outperform the KD variants in mean macro-F1 for all three students. The main conclusion is, therefore, that LCNet-0.5 is a strong compact architecture under the selected size–accuracy rule, not that KD is proven to be the cause of the observed compact-model performance.

The Raspberry Pi 3 Model B+ prototype demonstrates that the selected LCNet-0.5 model can be embedded in a local camera-to-display workflow, with an observed end-to-end latency of approximately 1.0 s per image. This result is useful for household or educational smart-bin interaction, but it is not a complete hardware benchmark. The reported latency includes image capture, preprocessing, local model execution, softmax, and LCD update; isolated inference latency, throughput, model load time, peak memory, active power, and energy per inference remain to be measured with dedicated instrumentation.

Several limitations remain. First, the KD evidence is incomplete: future work should train no-KD baselines under the same 100-epoch budget as the main split and run a full τ–α sensitivity grid. Second, the augmentation pipeline should be expanded to compare crop/flip against Mixup, CutMix, and minority-focused augmentation, with special attention to *trash* recall and F1. Third, the INT8 values reported here are size estimates only; post-training quantization and quantization-aware training should be evaluated for both accuracy and runtime. Fourth, the edge evaluation should be extended to a full benchmark covering inference-only latency, throughput, peak memory, power, and energy per inference on Raspberry Pi, Jetson-class devices, or microcontroller-class targets.

The RealWaste experiment already shows that zero-shot transfer from controlled TrashNet imagery to cluttered real-world imagery is difficult, while short fine-tuning substantially improves performance. This finding should guide future deployment: compact waste classifiers should be adapted with local data before use in new environments. Looking forward, the most important next step is, therefore, not only to tune KD, but also to combine compact architecture selection with domain adaptation, rigorous quantization evaluation, and instrumented hardware benchmarking.

## Data Availability

Publicly available datasets were analyzed in this study. The TrashNet dataset is available from its original repository (Thung and Yang, 2016; https://github.com/garythung/trashnet), and the RealWaste dataset is available from its original publication and repository (Single et al., 2023). This study uses the overlapping six-class waste-classification setting described in Section 5.
